# Cefmetazole-Induced Coagulopathy in a Patient With Vitamin K Deficiency: A Case Report With Literature Review

**DOI:** 10.7759/cureus.79540

**Published:** 2025-02-24

**Authors:** Shunsuke Nakamura, Natsuyo Shinohara

**Affiliations:** 1 Department of Emergency Medicine, Okayama Rosai Hospital, Okayama, JPN

**Keywords:** an n-methyltetrazole thiol (nmtt) group, cefmetazole (cmz), chronic vitamin k deficiency, coagulopathy, intra-abdominal infections

## Abstract

Cefmetazole (CMZ), a cephamycin antibiotic containing an N-methyltetrazolethiol (NMTT) group, is known to interfere with vitamin K metabolism, potentially causing coagulation disorders. We present the case of a 78-year-old man with chronic kidney disease who developed a coagulation disorder during CMZ administration, exacerbated by chronic vitamin K deficiency from prolonged enteral nutrition. In the context of chronic vitamin K deficiency precipitated by prolonged enteral nutrition for renal disease, the administration of CMZ-a medication that disrupts vitamin K metabolism and reduces coagulation factor activity through a mechanism similar to that of warfarin-led to abnormal blood coagulation test results. Clinical improvement followed the discontinuation of CMZ and vitamin K supplementation. This report highlights the importance of monitoring vitamin K status in patients receiving CMZ, particularly those with risk factors for deficiency, and reviews the relevant literature to highlight the mechanisms and management strategies for such complications.

## Introduction

Cefmetazole (CMZ) is widely used for the treatment of intra-abdominal infections and as perioperative prophylaxis. Its N-methyltetrazolethiol (NMTT) side chain can disrupt vitamin K metabolism by inhibiting vitamin K epoxide reductase, an enzyme essential for vitamin K recycling [1,2]. Vitamin K is a critical cofactor for the synthesis of coagulation factors II, VII, IX, and X. Critically ill patients receiving enteral nutrition with inadequate vitamin K levels are at increased risk of developing coagulation disorders [3]. Previous reports have noted coagulation abnormalities associated with CMZ, but the role of baseline vitamin K deficiency has not been thoroughly investigated [4]. Here, we present a detailed case and review the literature to further the understanding of this rare but significant complication.

## Case presentation

A 78-year-old man was brought to the emergency department because of hypothermia and circulatory failure. He had chronic kidney failure. Tracheal intubation and artificial respiration management were performed for his consciousness disorder and unstable hemodynamics. He was immediately admitted to the intensive care unit (ICU) and rewarmed. His rewarming was completed, but continuous hemodiafiltration (CHDF) was introduced for his anuria because of acute kidney injury caused by hypovolemia and hypothermia. He could not be extubated because of both sides’ recurrent laryngeal nerve paralysis. On day 5, we decided to convert CHDF to hemodialysis (HD) because his anuria had remained and his hemodynamics was stable. On day 9, a tracheotomy was performed and he exited ICU on day 12. Because he could not ingest any food even after exiting the ICU, enteral nutrients for the patients with chronic kidney disease (CKD) had been administered. Thereafter, we repeatedly performed antibiotic therapy: penicillin antibiotic or new quinolone-based antibacterial agent, to aspiration pneumonitis, cellulitis, and urinary tract infection as needed. On day 39, gastrostomy was performed because of the difficulty of ingestion and we used CMZ as the perioperative antibiotic. On the next day, he had a fever but we diagnosed it as localized peritonitis and continued to use the same antibiotic. As Table 1 shows the blood test on day 45 suddenly showed an abnormal value of the coagulability: prothrombin time-international normalized ratio (PT-INR) (3.25), activated partial thromboplastin time (APTT) (57.5 seconds).

**Table 1:** Laboratory data on day 45 The patient suddenly developed a prolonged prothrombin time-international normalized ratio (PT-INR 3.25) and activated partial thromboplastin time (APTT 57.5 seconds) on the 45th hospital day. WBC: white blood cell; RBC: red blood cell; Hb: hemoglobin; Ht: hematocrit; PLT: platelet; AST: aspartate aminotransferase; ALT: alanine aminotransferase; LD: lactate dehydrogenase; UN: urea nitrogen; Cre: creatinine; T. Bil: total bilirubin; CRP: C-reactive protein; Alb: albumin; PT: prothrombin time; INR: international normalized ratio; APTT: activated partial thromboplastin time; Fibg: fibrinogen; FDP: fibrin degradation product The standard unit of measurement for red blood cell count is 10^6^, while the standard unit of measurement for platelet count is 10^3^.

	Result	Units	Reference Values
White blood cell	7390	/μL	3300 - 8600
Red blood cell	3.00*10^6^	/μL	4.35 - 5.55*10^6^
Hemoglobin	8.3	g/dL	13.7 - 16.8
Hematocrit	27.5	%	40.7 - 50.1
Platelet	441*10^3^	/μL	155 - 348*10^3^
Aspartate aminotransferase	43	U/L	13 - 30
Alanine aminotransferase	13	U/L	10 - 42
Lactate dehydrogenase	153	U/L	124 - 222
Urea nitrogen	41.7	U/L	8 - 20
Creatinine	3.91	mg/dL	0.65 - 1.07
Total bilirubin	0.2	mg/dL	0.4 - 1.5
C-reactive protein	23.36	mg/dL	< 0.14
Sodium	137	mmol/L	138 - 145
Potassium	3.0	mmol/L	3.6 - 4.8
Chlorine	94	mmol/L	101 - 108
Albumin	2.2	g/dL	4.1 - 5.1
PT(%)	10	%	80 - 120
PT-international normalized ratio	3.25		0.9 - 1.1
Activated partial thromboplastin time	57.5	second	25 - 40
Fibrinogen	890	mg/dL	200 - 400
FDP	8.5	μg/mL	< 5.0
D-dimer	4.1	μg/mL	< 1.0

**Figure 1:**
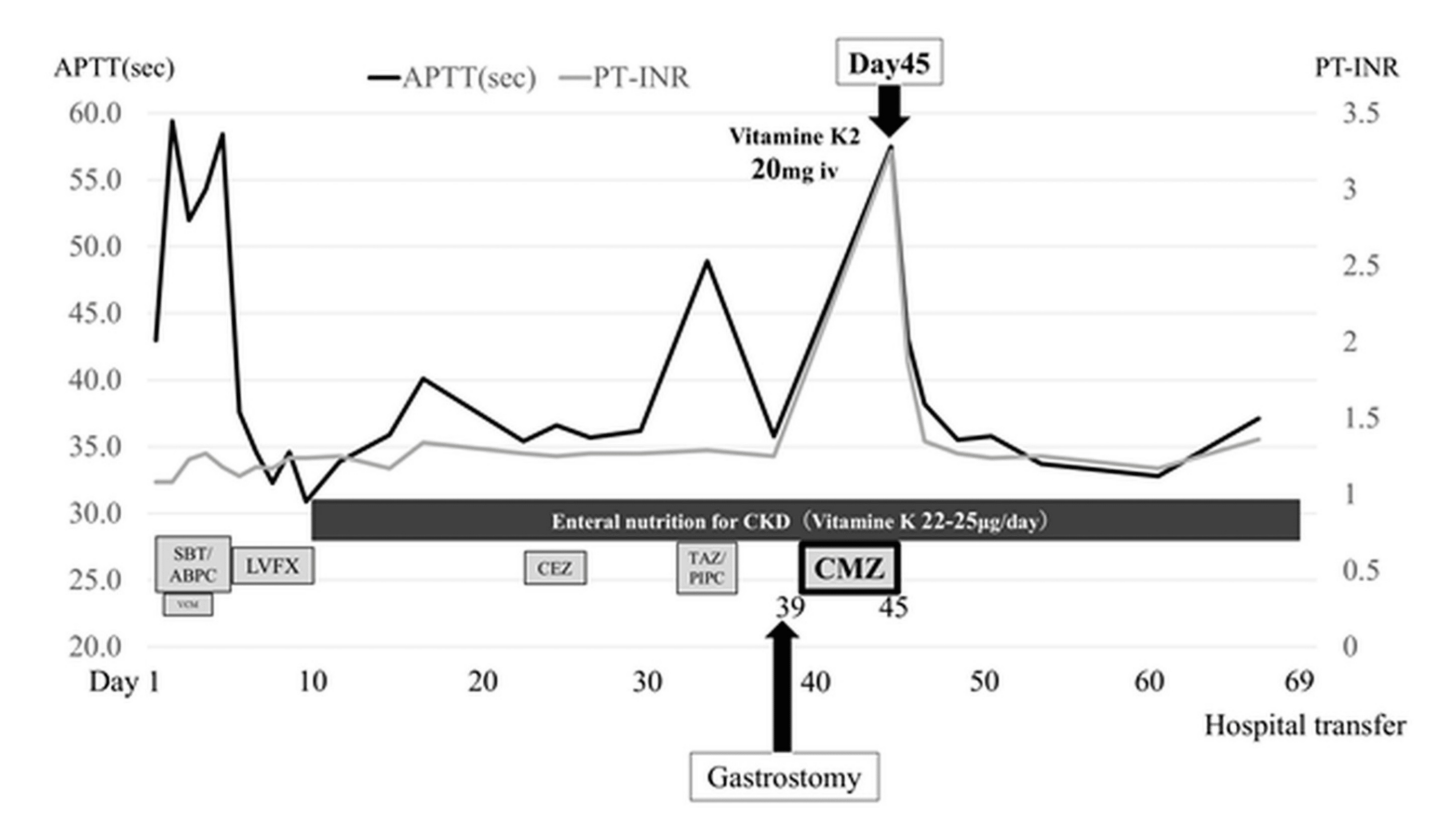
Timeline of the patient’s clinical course Timeline of the patient’s clinical course, including CMZ administration, onset of coagulation abnormalities, and response to vitamin K supplementation. SBT/ABPC: sulbactam/ampicillin; VCM: vancomycin; LVFX: levofloxacin; CEZ: cefazolin; TAZ/PIPC: tazobactam/piperacillin; CMZ: cefmetazole; CKD: chronic kidney disease; PT: prothrombin time; PT-INR: prothrombin time-international normalized ratio; APTT: activated partial thromboplastin time

His general appearance, however, was good, and disseminated intravascular coagulation (DIC) must have been negative because the other values relating to coagulability were not significantly abnormal: platelet (441,000/μL), fibrinogen (890 mg/dL), and D-dimer (4.1 μg/mL). The hepatobiliary disease was also negative because there were no abnormal findings suggesting decreased liver function in his blood test and computerized tomography. He did not take orally any anticoagulants and there were no other additional drugs except for CMZ. There were no findings suspecting the change of his intestinal microflora such as diarrhea or stool culture-positive. Then, we ruled the other differential diagnosis out and suspected that these results of the blood test were caused by the shortages of vitamin K because enteral nutrients for CKD, containing only 22-25 μg/day vitamin K (the recommended daily intake is 150 μg/day for Japanese) [1], had been administered for about a month. Protein induced by vitamin K absence or antagonist-II (PIVKA- II) highly increased to 19,792 mAU/mL, and we diagnosed this clotting disorder as the side effect of CMZ, inhibiting the metabolism of vitamin K, and the shortages of vitamin K because of the clinical course. Therefore, we decided to stop CMZ and administer vitamin K (20 mg) intravenously. The clotting disorder immediately improved and never occurred in our hospital after the intervention, and the patient was transferred to a rehabilitation hospital on day 69 (Figure 1).

## Discussion

**Figure 2:**
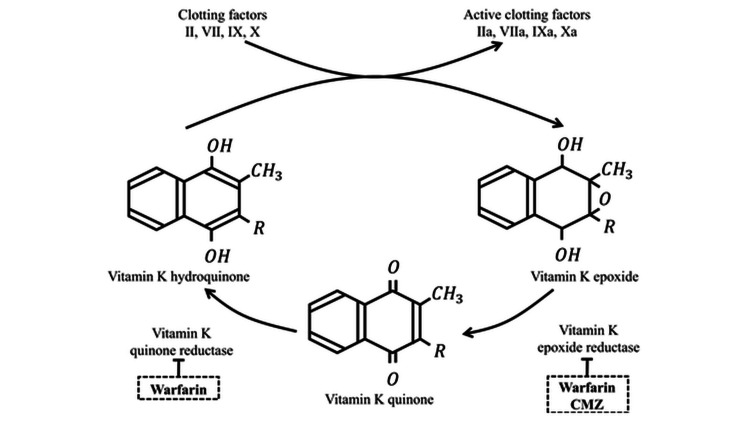
Mechanism of Vitamin K Metabolism Inhibition by Warfarin and Cefmetazole. Mechanism of Vitamin K metabolism inhibition by warfarin and cefmetazole. This figure was created by the authors based on the data in Kimura et al. [6].

The pathophysiology of CMZ-induced coagulopathy involves the inhibition of vitamin K-dependent coagulation factor synthesis: the NMTT group of CMZ inhibits vitamin K epoxide reductase, thereby inhibiting vitamin K recycling [5]. This mechanism is similar to the anticoagulant effect of warfarin (Figure 2).In our case, the symptoms were exacerbated by inadequate vitamin K intake from enteral nutrition.

Vitamin K deficiency is often overlooked in critically ill patients, especially those on special diets with limited content. Studies suggest that vitamin K intake below 1.0 µg/kg/day, as in this patient, may predispose to deficiency; active monitoring of coagulation parameters and procoagulant factor II levels is essential in high-risk patients receiving CMZ [1,4].

A survey of the literature reveals several reports of CMZ-induced coagulation abnormalities: Kodama et al. reported a similar case but did not emphasize baseline vitamin K intake [7]; Haba et al. reported an anticoagulant effect of CMZ without detailed nutritional assessment [8]; Nakano et al, emphasized the importance of vitamin K supplementation for critically ill patients receiving CMZ [3]. These findings emphasize the importance of individualized nutritional strategies and early recognition of coagulation abnormalities in at-risk patients.

The patient's initial nutritional status remained uncertain; however, following admission, he required dialysis due to significant renal impairment. He had been receiving long-term enteral nutrition for renal disease. A comprehensive review of the vitamin K content in all nutritional supplements administered post-admission revealed a noticeable deficiency in intake. Management of CMZ-induced coagulopathy of this high-risk patient includes prompt drug discontinuation and vitamin K administration. Prophylactic vitamin K supplementation should be considered in high-risk patients. Clinicians should strongly suspect coagulopathy in chronically ill or long-term enterally fed patients taking CMZ. While the limitations of this case report preclude a comprehensive discussion, they provide an opportunity to explore the efficacy of specific nutritional administration protocols for critically ill patients requiring long-term enteral nutrition.

## Conclusions

This case highlights the increased risk for critically ill patients requiring chronic enteral nutrition, emphasizing the essential role of vitamin K in coagulation and the potential for CMZ to cause significant abnormalities in deficient states. Prospective monitoring of coagulation parameters and proactive supplementation may help prevent severe complications. Further studies are needed to establish standardized protocols for managing and preventing CMZ-induced coagulopathy in vulnerable populations.
